# Clinical effectiveness of care managers in collaborative primary health care for patients with depression: 12- and 24-month follow-up of a pragmatic cluster randomized controlled trial

**DOI:** 10.1186/s12875-022-01803-x

**Published:** 2022-08-09

**Authors:** Sandra af Winklerfelt Hammarberg, Cecilia Björkelund, Shabnam Nejati, Maria Magnil, Dominique Hange, Irene Svenningsson, Eva-Lisa Petersson, Malin André, Camilla Udo, Nashmil Ariai, Lars Wallin, Carl Wikberg, Jeanette Westman

**Affiliations:** 1 Division of Family Medicine and Primary Care, Department of Neurobiology, Care Sciences and Society, Karolinska Institutet, Alfred Nobels Allé 23, 141 52 Huddinge, Stockholm, Sweden; 2 Academic Primary Health Care Centre, Region Stockholm, Stockholm, Sweden; 3 Department of Public Health and Community Medicine, Primary Health Care, Institute of Medicine, Sahlgrenska Academy, University of Gothenburg, Gothenburg, Sweden; 4 Region Västra Götaland, Närhälsan Research and Development Primary Health Care, Gothenburg, Sweden; 5 Department of Public Health and Caring Sciences – Family Medicine and Preventive Medicine, Uppsala University, Uppsala, Sweden; 6 School of health and Welfare, Dalarna University, Falun, Sweden; 7 Division of Health Care Science, Marie Cederschiöld University, Stockholm, Sweden; 8 Center for Clinical Research Dalarna, Uppsala University, Falun, Sweden; 9 Division of Nursing, Department of Neurobiology, Care Sciences and Society, Karolinska Institutet, Huddinge, Sweden

**Keywords:** Care manager, Collaborative care, Depression, Primary health care, Quality of life, Symptom severity, Confidence in care

## Abstract

**Background:**

In previous studies, we investigated the effects of a care manager intervention for patients with depression treated in primary health care. At 6 months, care management improved depressive symptoms, remission, return to work, and adherence to anti-depressive medication more than care as usual. The aim of this study was to compare the long-term effectiveness of care management and usual care for primary care patients with depression on depressive symptoms, remission, quality of life, self-efficacy, confidence in care, and quality of care 12 and 24 months after the start of the intervention.

**Methods:**

Cluster randomized controlled trial that included 23 primary care centers (11 intervention, 12 control) in the regions of Västra Götaland and Dalarna, Sweden. Patients ≥18 years with newly diagnosed mild to moderate depression (*n* = 376: 192 intervention, 184 control) were included. Patients at intervention centers co-developed a structured depression care plan with a care manager. Via 6 to 8 telephone contacts over 12 weeks, the care manager followed up symptoms and treatment, encouraged behavioral activation, provided education, and communicated with the patient’s general practitioner as needed. Patients at control centers received usual care. Adjusted mixed model repeated measure analysis was conducted on data gathered at 12 and 24 months on depressive symptoms and remission (MADRS-S); quality of life (EQ5D); and self-efficacy, confidence in care, and quality of care (study-specific questionnaire).

**Results:**

The intervention group had less severe depressive symptoms than the control group at 12 (*P* = 0.02) but not 24 months (*P* = 0.83). They reported higher quality of life at 12 (*P* = 0.01) but not 24 months (*P* = 0.88). Differences in remission and self-efficacy were not significant, but patients in the intervention group were more confident that they could get information (53% vs 38%; *P* = 0.02) and professional emotional support (51% vs 40%; *P* = 0.05) from the primary care center.

**Conclusions:**

Patients with depression who had a care manager maintained their 6-month improvements in symptoms at the 12- and 24-month follow-ups. Without a care manager, recovery could take up to 24 months. Patients with care managers also had significantly more confidence in primary care and belief in future support than controls.

**Trial registration:**

ClinicalTrials.gov identifier: NCT02378272. Submitted 2/2/2015. Posted 4/3/2015.

**Supplementary Information:**

The online version contains supplementary material available at 10.1186/s12875-022-01803-x.

## Background

More than 300 million people worldwide have depression—an estimated 4.4% of the population—which makes it the world’s most common mental disorder [1, 2]. Most people with depression seek care in primary health care [2], and many countries have made efforts to improve the structure and organization of care for these patients [3].

Evidence suggests that single interventions, such as increased screening for depression and education for general practitioners and nurses, do not improve care or outcomes for patients with depression [4]. On the other hand, organizational changes to improve care coordination significantly improve depression outcomes [5, 6]. In coordinated care programs, primary care professionals with different areas of expertise work together for the benefit of patients. Typical components include a structured management plan for patients with scheduled patient follow-ups, enhanced interprofessional communication, introduction of decision support systems, and simplified guidelines. A key component of this enhanced care is the care manager, often a specially educated nurse, who acts as the central coordinator of patient care [7]. Care managers can provide a variety of support and feedback to patients, maintain contact with patients’ physicians, and contact other care or service providers as needed.

Studies on collaborative care for patients with depression have been carried out in the United States, Canada, and Europe [5]. A meta-analysis of 79 randomized controlled studies [5] showed robust and clear evidence that care manager interventions improve care for primary care patients with depression [5]. Collaborative care was more effective in reducing depressive symptoms than usual care in the short term (3–6 months), medium term (7–12 months), and long term (13–24 months) [5]. However, as primary care conditions vary between countries, and no such interventions had been tested in Swedish primary care, we set out to evaluate the clinical effectiveness of care managers in collaborative care for patients with depression in this setting. We designed and implemented PRIM-CARE, a cluster randomized controlled trial (randomized at the primary care center level) in two regions of Sweden.

At the 6-month follow-up, the results of PRIM-CARE were similar to those of the international studies [5, 8]. Care managers had positive effects on depressive symptoms, remission, return to work, and guideline-concordant use of antidepressants. Like the care manager studies from other countries, PRIM-CARE showed that care managers were cost-effective [9, 10]. However, the long-term outcomes of PRIM-CARE have not yet been evaluated.

### Aim

To compare the 12- and 24-month effects of care management and care as usual for patients with mild to moderate depression treated in primary care. Outcomes included depressive symptoms, remission from depression, quality of life, self-efficacy, confidence in care, and quality of care from a patient perspective.

## Methods

**Fig. 1:**
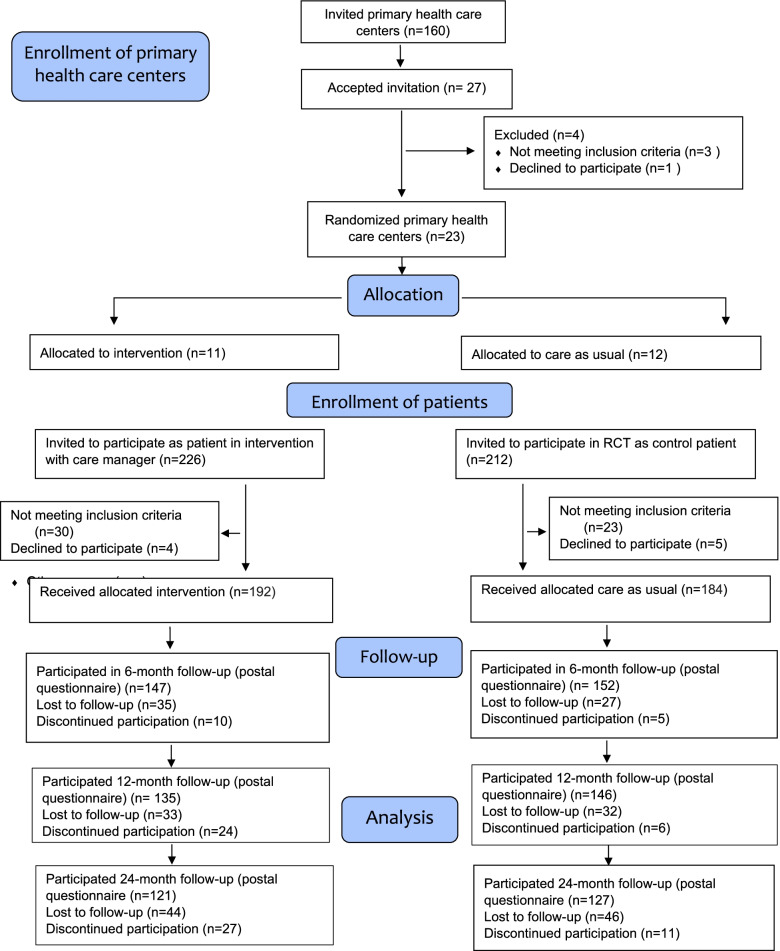
Consort flow chart of the PRIM-CARE randomized controlled trial and follow-ups from baseline to 24 months

PRIM-CARE was a pragmatic cluster randomized controlled trial that compared a 12-week nurse care-manager program with treatment as usual for patients with depression [8]. The methods have been described in greater detail in previous publications [8, 10]. In brief, the study was carried out at 23 primary care centers (11 intervention and 12 control) in the regions of Västra Götaland and Dalarna, Sweden, starting in 2014. Centers were eligible for inclusion unless they already had a care manager or the equivalent of a care manager. The centers were located in rural and urban areas of varied socioeconomic status. Figure 1 presents a flow chart of the trial.

### Population

Patients at the participating primary care centers who met the eligibility criteria were invited to take part in the study. Patients were included if they were ≥ 18 years, newly diagnosed (< 1 month) with depression (International Classification of Diseases and Other Health Problems, 10th edition: diagnostic codes F32 and F33) [11], and the depression was mild or moderate (< 35 points on the Montgomery-Asberg Depression Rating Scale) [12, 13]. Patients who had recovered from previous episodes of depression could be included in the study. Patients were excluded if they had a current diagnosis of cognitive impairment (e.g., dementia), bipolar disorder, psychosis, or substance use disorder or were unable to speak or read Swedish.

### Outcomes

Outcomes in the PRIM-CARE study were measured at baseline, 3, 6, 12, and 24 months.

*Depressive symptoms* were measured with the Montgomery-Asberg Depression Rating Scale - Self-assessment (MADRS-S) [12, 13]. MADRS-S has nine items and asks about symptoms during the last 3 days. Total scores range from 0 to 54. A total of 0 to 12 points indicates no or very mild symptoms of depression; 13 to 19 points, mild symptoms; 20 to 34 points, moderate symptoms; and 35 to 54 points, severe symptoms.

*Fifty percent reduction in depressive symptoms* as measured with MADRS-S scores from baseline to each follow-up occasion was also investigated.

*Remission from depression* was operationalized as a score of < 12 points on MADRS-S, which indicates no or very mild symptoms of depression.

*Quality of life* was measured with the EuroQol five Dimension 3 L scale, English tariff (EQ-5D-3L) [14, 15]. The EQ-5D-3L is a three-level version of the EQ-5D health index. It measures health-related quality of life and has two sections. In the first section, respondents select one of three levels (no problems, some problems, extreme problems) that best matches their health-related quality of life in the areas of mobility, self-care, everyday activities, pain/discomfort, and anxiety/depression.

*Self-efficacy*, *confidence in care*, and *quality of care from a patient perspective* were assessed at 24 months via a study-specific questionnaire. The English translation of the questionnaire is presented in Supplemental material 1. The 20 items on the questionnaire were inspired by items on patient questionnaires about self-efficacy [16–19] and an instrument about quality of care from a patient perspective [20]. The study-specific questionnaire was not validated. Response alternatives for the statements about self-efficacy and confidence in care were provided on a 5-level Likert scale that ranged from *not at all confident* to *completely confident.* Responses were dichotomized in the analysis (*not at all confident* to *moderately confident* versus *very confident* to *completely confident*). Responses to the statements about quality of care from a patient perspective were provided on a 5-level Likert scale that ranged from *not at all true* to *completely true*. Responses to this item were also dichotomized in the analysis (*not at all true* to *moderately true* versus *very true* to *completely true*).

Use of *antidepressant medication* (Y/N) was an outcome in the analyses because a goal of care management was to support patients in adhering to antidepressant treatment [8]. According to national guidelines, once antidepressant use is initiated, patients should continue to use the medication for at least 6 months [21].

### Other variables

Data on patient background variables were collected at baseline, including *age*, *sex*, *working* (full-time, other [25–75% of full time]), *marital status* (cohabiting, single), *born outside Nordic country* (Y/N), *educational level* (primary education, secondary education, university or college education), *leisure-time physical activity* (sedentary Y/N), *smoking* (Y, sometimes/No), *alcohol* at least once a week (Y/N), *sick leave last year* (Y/N), and *on sick leave at baseline* (Y/N).

*Psychotherapy* use was also monitored during the study period. Starting at the 3-month follow-up, the patients reported whether they had had contact with a psychologist or psychotherapist since baseline (or the previous follow-up), and if so, how often. At the 3-month follow-up, these self-reported data were complemented with data from electronic patient records. Psychotherapy use (Y/N) was treated as a potential confounder in the analysis.

### Randomization

A total of 23 primary care centers were included and divided into two strata, rural (*n* = 12) and urban (*n* = 11) [8]. The centers in each stratum were then divided into six blocks of two centers each. One center in each block was randomized to the intervention group (*n* = 11) and one to the control group (*n* = 12) (Fig. 1) [8]. Randomization was performed by a statistician who was not involved in the study and who was blinded to the identity of the primary care centers. Cluster randomization was chosen to avoid treatment contamination between the intervention and control groups [22], which can occur if patients are individually randomized. This design is common in studies of organizational changes [23, 24].

### Intervention

One nurse at each intervention center worked approximately 20 to 25% of full-time as care manager. Care managers received a total of 5 days of training in delivering the intervention according to the protocol [8]. General practitioners and managers at the intervention centers also received 2 days of training. Every other month during the study period, personnel from the region and study personnel held in-person support meetings for care managers. A research assistant from the study group visited the intervention centers weekly to provide additional support for personnel and monitor adherence to the protocol.

The intervention was 12 weeks long. The care manager first met the patient for a one-hour meeting to develop an individual care plan. The care manager followed up the care plan with a phone call at 2, 4, 6, 8, and 12 weeks. The purpose of these calls was to monitor depressive symptoms with a rating scale (MADRS-S), encourage behavioral activation, and support adherence to medication and recovery. Care managers stayed in regular contact with the patient’s general practitioner, therapist, and other care providers to inform them about changes that required attention (e.g., potential side effects of medication, new or worsening symptoms, or failure to respond to treatment). Patients could also call the care manager as needed. At the end of the intervention, the care manager and patient developed an individual relapse prevention plan.

### Control

Patients at the control primary care centers received usual care, which did not include the intervention (i.e., a care manager). The Swedish National Board of Health and Welfare provides guidelines for treating depression and anxiety in primary care [21]. In brief, these guidelines state that patients should receive a rapid follow-up appointment after diagnosis with depression, as well as ongoing easy access to the general practitioner. Recommended treatment for depression includes cognitive behavioral or interpersonal therapy and/or antidepressant medication [21]. A research assistant from the study group visited control centers regularly to monitor adherence to the protocol and collect data about routine care from electronic patient records.

### Enrollment of patients, diagnostic procedure, and data collection

During patients’ primary care center appointment for depressive symptoms, general practitioners invited the patients to take part in the study. Patients received verbal and written information about the study and provided written informed consent to participate. They received an appointment with the care manager (intervention centers) or a study nurse (control centers), who used the depression module of PRIME–MD [25] to confirm the general practitioner’s diagnosis of mild to moderate depression. At the intervention centers, care managers collected baseline data, and at control centers, a study nurse collected them. Follow-up data were gathered with questionnaires sent via regular mail 6, 12, and 24 months after the start of the study.

### Statistical analyses

Continuous variables, such as age and symptom severity, were analyzed with the independent sample *t*-test or Mann-Whitney U test. Categorical variables were described as numbers and percentages and analyzed with Pearson’s chi-square test. Mean intra-individual change in depressive symptoms and quality of life in the intervention and control group were compared with mixed model analysis with repeated measures, adjusted for the type of primary care center (sparse vs medium-high patient inclusion rate) and the patient’s age, sex, educational level, use of antidepressant medication, and variable-related scores at baseline [26]. Statistical significance was set at *P* < 0.05. No multiple adjustments were considered. Because of sparse data from some of the primary care centers, we did not adjust for the cluster randomization.

Power calculations are shown in detail in the article that describes the 3- and 6-month results of PRIM-CARE [8]. The statistical analyses were carried out with SPSS, version 25, and SAS, version 9.4.

### Ethical considerations

The Regional Ethical Review Board in Gothenburg approved the study on 2 January 2014 (Dnr 903–13) and the 24-month follow up on 27 July 2018 (T598–18). Complementary approval for the Dalarna portion of the study was received from the Regional Ethical Review Board in Gothenburg on 7 January 2015 (T975–14). The study was carried out in accordance with the World Medical Association Declaration of Helsinki [27].

The heads of primary care in Region Västra Götaland and Dalarna County provided written permission to conduct the study, as did the manager of each participating primary care center. Participating personnel provided verbal informed consent. After receiving verbal and written information about the study and before they were included in the study, participating patients provided written informed consent.

The study was registered on 2 February 2015 at ClinicalTrials.gov, identifier NCT02378272.

## Results

Of the 192 patients in the intervention group, 135 (70%) returned the postal questionnaires at the 12-month follow-up and 121 (63%) at the 24-month follow-up (Fig. 1). A similar pattern was seen in the control group. Of the 184 patients, 146 (79%) returned the postal questionnaires at the 12-month follow-up and 127 (69%) at the 24-month follow-up.

Supplemental Table 1 shows the background characteristics of the patients in the intervention group and control group. There were no significant differences between the intervention and control groups at baseline, between the intervention and control groups at 12 months (after dropout), or between the intervention and control groups at 24 months (after further dropout) (Supplemental Table 1). The background characteristics of the study population are described in detail in a previous publication [8]. There were no significant differences in psychotherapy use at any follow-up or across the total study period (0–24 months: intervention group 47%, control group 49%, *P* = 0.75).

**Fig. 2:**
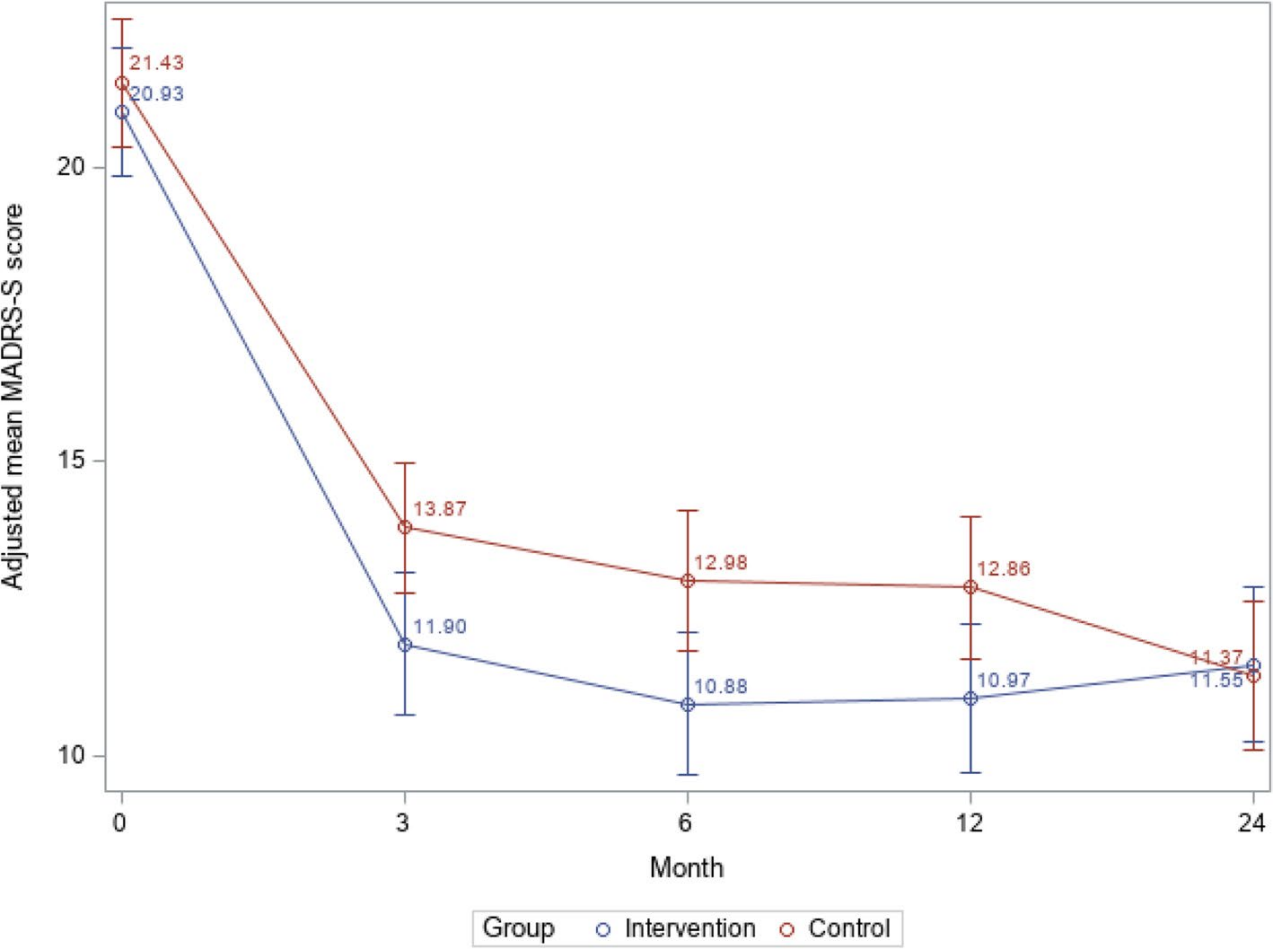
Depressive symptoms^1^ from baseline to 24 months in the intervention group and control group Abbreviations: *MADRS-S* Montgomery-Asberg Depression Rating Scale – Self rating version ^1^ Measured with MADRS-S. . P12 months=0.02; P24 months=0.83

At the 12-month follow-up, there was a statistically significant difference in the level of depressive symptoms in the intervention and control group. The intervention group had less severe depressive symptoms than the control group (95% confidence interval (CI): − 3.50 to − 0.26; *P* = 0.02) (Fig. 2). However, at the 24-month follow-up, symptom severity in the control group had reached the same level as in the intervention group (95% CI: − 1.53 to 1.90; *P* = 0.83) (Fig. 2).

Two analyses were added to further explore clinically relevant outcomes often examined in depression research: 50% reduction in depressive symptoms and the proportion of patients in remission. There were significant differences in 50% reductions in depressive symptoms (measured with MADRS-S scores) between the intervention and control groups at 6 months. Reductions continued but were not significant between groups at 12 or 24 months (Table 1). There were significant differences in the proportion of patients in remission (MADRS-S score < 12 points) at 6 months but not at 12 or 24 months (Table 1). Almost half the patients who responded at follow-up continued to use antidepressants at 12 months (41.5% in the intervention group and 49.3% in the control group), and more than a third continued to use such medication at 24 months (36.4% in the intervention group and 38.6% in the control group) (Table 1). There was no statistically significant difference in antidepressant use between the groups on either occasion (Table 1).

**Table 1:** Reduction in depressive symptoms, remission from depression, and anti-depressant medication at 6, 12 and 24 months

Variables	6 months		***p***	12 months		***p***	24 months		***p***
	Intervention n (%)	Control n (%)		Intervention n (%)	Control n (%)		Intervention n (%)	Control n (%)	
Reduction > 50% MADRS-S	76 (52.1)	60 (39.5)	0.029	66 (49.6)	66 (45.8)	0.53	63 (52.5)	59 (46.5)	0.34
Remission from baseline MADRS< 12	83 (66.9)	67 (49.3)	0.004	74 (64.3)	70 (54.3)	0.11	66 (64.1)	67 (58.8)	0.42
Antidepressant medication (yes)	75 (51.0)	92 (60.5)	0.098	56 (41.5)	72 (49.3)	0.19	44 (36.4)	49 (38.6)	0.72

*Abbreviations: MADRS-S* Montgomery-Asberg Depression Rating Scale-self-rating version

**Fig. 3:**
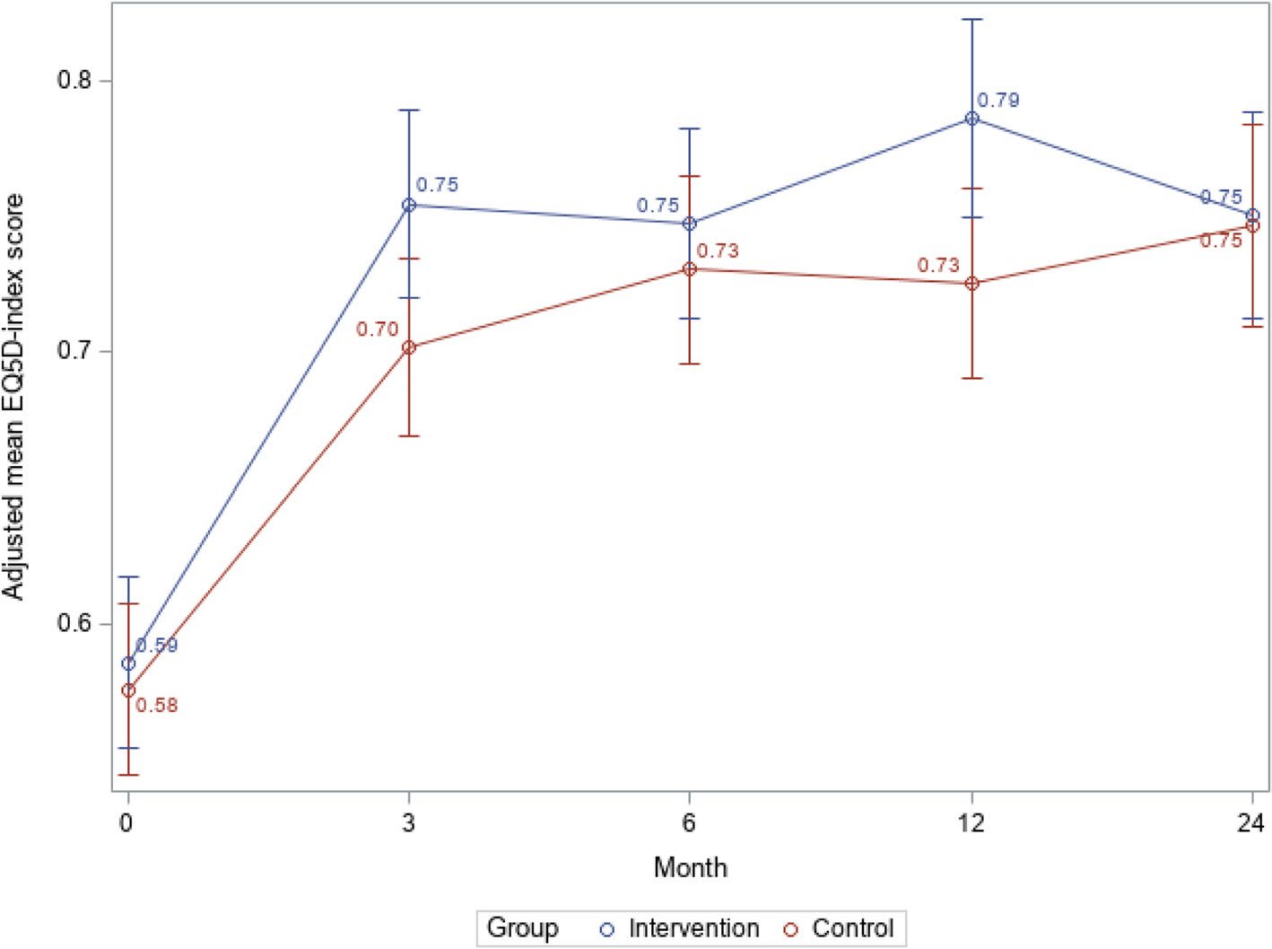
Quality of life^1^ from baseline to 24 months in the intervention group and control group Abbreviations: *EQ-5D* EuroQol five Dimension 3 L scale ^1^ Measured with EQ-5D. P12 months=0.01; P24 months=0.88

The intervention group reported significantly higher quality of life than the control group at the 12-month follow-up (95% CI: 0.01 to 0.11; *P* = 0.01) (Fig. 3). However, at the 24-month follow-up, quality of life in the control group had reached the same level as in the intervention group (95% CI: − 0.05 to 0.05; *P* = 0.88) (Fig. 3).

At the 24-month follow-up, there were significant differences between the two groups in responses to statements about confidence in care on the study-specific questionnaire. Specifically, 53% of patients in the intervention group and 38% in the control group had confidence that they would be able to get information about their illness/symptoms from the primary care center (*P* = 0.02), and 51% in the intervention group and 40% in the control group had confidence that they would be able to get professional emotional support (*P* = 0.05).

Responses to one of the three statements about quality of care from a patient perspective also differed significantly between the two groups. A total of 49% of the patients in the intervention group and 36% of the patients in the control group agreed that they had received good information about their treatment (*P* = 0.03). There were no significant differences in responses to statements about self-efficacy.

## Discussion

### Summary of main findings

This long-term follow-up study found that primary care patients with depression who had care managers maintained their previous improvements [8] in depressive symptoms 12 and 24 months after the care manager intervention. Although depressive symptoms also improved over time in patients who received usual care, a year after the intervention those patients still had statistically significantly more severe depressive symptoms than the patients who were assigned a care manager. Twenty-four months after the intervention, levels of depressive symptoms were similar in the two patient groups. A different pattern emerged when we investigated 50% symptom reduction and remission. Although those analyses showed significant differences at the 6-month follow-up, differences between the patients who did and did not have a care manager were not significant at either the 12-month follow-up or the 24-month follow-up. Patients with care managers had significantly better quality of life after 12 but not 24 months. We observed no differences in self-efficacy at either follow-up, but the findings suggested that care managers improved patients’ confidence in care and quality of care from a patient perspective.

### Comparison with previous studies

Numerous previous studies and systematic reviews have evaluated the long-term effects of collaborative care for primary care patients with depression, but to the best of our knowledge, there have been no such studies in Swedish primary care. Our findings about depressive symptoms in the patients assigned a care manager are broadly consistent with the results of previous meta-analyses [5, 28]. Those analyses found that collaborative care models are more effective than usual care in treating depression for follow-up periods of up to 24 months [5, 28].

As in our study, one of the previous systematic reviews found that collaborative care can lead to better quality of life than usual care [5]. However, the quality-of-life outcome in that study was more nuanced than our general measure of this variable. The researchers in the previous study found that patients who received collaborative care had better long-term mental health quality of life than those who received usual care. There was less evidence, though, that collaborative care led to better physical quality of life. Another meta-analysis of patients with depression and other comorbid chronic illnesses concluded that collaborative care is more effective than usual care in reducing illness burden and improving physical outcomes [29], which could be interpreted as indicative of improved physical quality of life.

Outcomes of depression care can be measured in several ways, such as reduced depressive symptoms, increased quality of life, and return to work. But in the long-term, the most important outcomes may be recovery and relapse prevention [30]. As previously noted, like other collaborative care interventions [5, 28], PRIM-CARE improved recovery [8]. However, there were no significant differences in remission (defined as a score of < 12 points on MADRS-S) between patients in the intervention group and the control group in the long-term (at either 12 or 24 months). Thus, it was not possible to determine whether the intervention prevents relapse or not. This is noteworthy because in PRIM-CARE, care managers and patients co-developed a relapse prevention plan. Relapse prevention plans are one of four strategies identified in a previous systematic review of strategies used in collaborative care interventions to prevent relapse in patients with depression [30]. That study, however, did not examine the effectiveness of the four strategies.

As in our study, previous systematic reviews found evidence that care managers result in greater patient satisfaction than usual care [5]. For instance, we found that patients with care managers had significantly more confidence that they would be able to get professional emotional support and adequate information from the primary care center, which potentially indicates a readiness to contact primary care in case of possible relapse.

### Strengths and limitations

This study had several strengths. One was randomization at the primary care center level, which is appropriate for evaluating organizational changes [23, 24]. Another strength was the inclusion of primary care centers from two different regions of Sweden and from both rural and urban areas of varying socioeconomic status, which could increase the generalizability of the study findings. The use of validated instruments (MADRS-S, EQ5D-3 L) helped prevent bias. The long-term nature of the follow-up was also a strength, as it is important to evaluate organizational changes over long periods of time [24].

The study also had several limitations, including loss to follow-up, especially at 24 months. The loss was similar in the intervention group and the control group. Nevertheless, patients lost to follow-up in either or both groups could differ in important ways from those who were not lost to follow-up. Another limitation is that the cluster design made it impossible to blind patients or health care center staff to intervention status. Furthermore, although cluster effects are possible in cluster randomized controlled trials, we were not able to adjust for these effects, as data from some of the primary care centers were sparse. However, we adjusted for the type of primary care center (sparse vs medium-high patient inclusion rate). The questionnaire on self-efficacy and patient satisfaction with care was unvalidated, which makes it challenging to interpret the meaning of the findings that are based on responses to that questionnaire. We have therefore interpreted those findings with extra caution.

## Conclusions

This study shows patients with depression who had a care manager maintained the recovery and improvements in quality of life they had achieved at 6 months at the 12- and 24-month follow-ups. Without a care manager, however, recovery could take up to 24 months. Patients who were assigned a care manager also had significantly more confidence in primary care and belief in future support than controls.

## Supplementary Information

**Additional file 1.**

**Additional file 2: Supplemental Table 1.** Background characteristics of the study group at baseline, at 12 months (after dropout), and at 24 months (after further dropout). These data were measured once, at baseline.

## Data Availability

The dataset supporting the conclusions of this article were gathered as part of the PRIM-CARE study, ClinicalTrials.gov identifier: NCT02378272 submitted February 2, 2015 and posted 3/4/2015. The datasets generated and/or analyzed during the current study are not publicly available because of Swedish legislation but are available from the corresponding author on reasonable request.

## Abbreviations

ICD: International Classification of Diseases
MADRS-S: Montgomery-Asberg Depression Rating Scale - Self-assessment
EQ-5D: EuroQol five Dimension Scale
Y/N: Yes/no
