# Supplementary material for: Clinical effectiveness of care managers in collaborative primary health care for patients with depression: 12- and 24-month follow-up of a pragmatic cluster randomized controlled trial
Source: BMC Prim Care. 2022 Aug 9;23:198. doi: 10.1186/s12875-022-01803-x (PMC9361666; doi:10.1186/s12875-022-01803-x)
Supplement: Supplementary file 1 — Additional file 1. [file 12875_2022_1803_MOESM1_ESM.pdf]

SELF-EFFICACY/ CONFIDENCE IN OWN ABILITY

English version, translation from Swedish

Below are some questions about your confidence and the support you received from the health care center during your depression. Next to each question, mark an X in the box that you think best matches your degree of confidence.

**How confident are you that you can do the following things that can reduce or prevent symptoms of depression?**	**Not at all confident 0**	**Somewhat confident 1**	**Moderately confident 2**	**Very confident 3**	**Completely confident 4**
1. If you should feel depressed –can you then influence your feeling through activity?					
2. If you should feel anxiety – can you then influence your feeling through activity?					
3. If you should feel stressed, can you then influence your feeling through activity?					
4. If you think of yourself as inferior or that you are not good [enough] – can you affect the thought through activity?					
**How confident are you that you know:**	**Not at all confident 0**	**Somewhat confident 1**	**Moderately confident 2**	**Very confident 3**	**Completely confident 4**
5. . . . what you can do if you get sleep difficulties (for example sleep too much or too little)					
6. . . . what you could do to feel better					
7. . . . when you should contact or visit health care providers about your illness/symptoms					
8. . . . how to get staff at the primary care center to understand your problems					
**How confident are you that you can . . .**	**Not at all confident 0**	**Somewhat confident 1**	**Moderately confident 2**	**Very confident 3**	**Completely confident 4**
9. . . . maintain your usual social activities					
10. . . . maintain your usual activities at home					
11. . . . maintain your usual activities outside the home					
12. . . . overcome obstacles and focus on opportunities					
13. . . . handle difficult or new tasks					
14. . . . cope with setbacks in certain demanding situations					
15. . . . get professional emotional support if you need it					

16. . . . handle feelings of loneliness					
17. . . . get information about your illness/symptoms from the primary care center					
**How well are the statements below in accordance with your situation**	**Not at all true**	**Only partly true**	**Moderately true**	**Very true**	**Completely true**
18. I received good information about my illness/my symptoms					
19. I received good information about what treatment would be like					
20. I felt involved in decisions about my treatment					
