# Supplementary material for: Clinical effectiveness of care managers in collaborative primary health care for patients with depression: 12- and 24-month follow-up of a pragmatic cluster randomized controlled trial
Source: BMC Prim Care. 2022 Aug 9;23:198. doi: 10.1186/s12875-022-01803-x (PMC9361666; doi:10.1186/s12875-022-01803-x)
Supplement: Supplementary file 2 — Additional file 2: Supplemental Table 1. Background characteristics of the study group at baseline, at 12 months (after dropout), and at 24 months (after further dropout). These data were measured once, at baseline. [file 12875_2022_1803_MOESM2_ESM.pdf]

Supplemental table 1. Background characteristics of the study group at baseline, at 12 months (after dropout), and at 24 months (after further dropout). These data were measured once, at baseline.

	**Study group at baseline**			**Study group at the 12-month follow-up (after dropout)**			**Study group remaining at the 24-month follow-up (after further dropout)**		
Background characteristics measured at baseline	Intervention n=192	Control n=184	P^1	Intervention n=135	Control n=146	P^2	Intervention n=121	Control n=127	P^3
Age, mean (SD)	40.8 (15.0)	41.6 (15.4)	0.60	42.1 (14.2)	42.3 (15.4)	0.91	42.9 (14.3)	42.5 (14.8)	0.85
Sex n (%)									
Female	131 (68.2)	137 (74.5)	0.18	95 (70.4)	111 (76.0)	0.28	87 (71.9)	97 (76.4)	0.42
Male	61 (31.8)	47 (25.5)		40 (29.6)	35 (24.0)		34 (28.1)	30 (23.6)	
Working n (%)									
Full-time	157 (87.7)	149 (87.6)	0.99	113 (88.3)	120 (87.6)	0.86	103 (89.6)	102 (85.7)	0.37
Other (25%-75%)	22 (12.3)	21 (12.4)		15 (11.7)	17 (12.4)		12 (10.4)	17 (14.3)	
Marital status, n (%)									
Cohabiting	124 (65.3)	123 (66.8)	0.75	90 (66.7)	97 (66.4)	0.97	82 (68.9)	91 (71.7)	0.64
Single	66 (34.7)	61 (33.2)		45 (33.3)	49 (33.6)		37 (31.1)	36 (28.3)	
Born Outside Nordic country n (%)	23 (12)	27 (14.8)	0.44	11 (8.1)	18 (12.4)	0.24	9 (7.4)	15 (11.9)	0.24
Educational level n (%)									
Primary- Secondary	120 (62.8)	117 (63.9)	0.82	84 (62.2)	91 (62.3)	0.99	75 (62.0)	78 (61.4)	0.93
University or college	71 (37.2)	66 (36.1)		51 (37.8)	55 (37.7)		46 (38.0)	49 (38.6)	
Leisure-time physical activity, Sedentary n (%)	25 (13.1)	33 (17.9)	0.20	17 (12.6)	25 (17.1)	0.29	16 (13.2)	22 (17.3)	0.37
Smoking yes+sometimes n (%)	45 (23.6)	56 (30.4)	0.13	25 (18.5)	37 (25.3)	0.17	24 (19.8)	31 (24.4)	0.39
Alcohol at least once a week n (%)	75 (39.5)	74 (40.2)	0.88	50 (37.6)	58 (39.7)	0.72	50 (41.7)	53 (41.7)	0.99
Sick leave n (%)									
Sick leave last year, yes	83 (43.2)	66 (35.9)	0.15	59 (43.7)	53 (36.3)	0.21	54 (44.6)	46 (36.2)	0.18
On sick leave at baseline	93 (48.4)	94 (51.1)	0.61	68 (50.4)	70 (47.9)	0.69	61 (50.4)	63 (49.6)	0.90
Depressive symptoms, MADRS-S m (SD)	20.8 (7.2)	22.0 (7.1)	0.12	20.5 (7.5)	21.7 (7.0)	0.14	20.1 (7.1)	21.6 (6.9)	0.09

Antidepressant medication, yes n (%)[4]	101 (52.6)	115 (62.5)	0.05	56 (41.5)	72 (49.3)	0.19	44 (36.4)	49 (38.6)	0.72
Quality of life, EQ-5D-3L m (SD)	0.58 (0.24)	0.56 (0.25)	0.41	0.58 (0.25)	0.57 (0.25)	0.86	0.59 (0.25)	0.55 (0.25)	0.27

Abbreviations: m: mean; SD: standard deviation; MADRS-S: Montgomery-Asberg Depression Rating Scale-self-rating version; EQ-5D-3L: EuroQol five Dimension 3 L scale, English tariff

[1] *P* for the difference between background characteristics in the intervention and control group at baseline.

[2] *P* for the difference between background characteristics in the intervention and control group followed up at 12 months (after dropout).

[3] *P* for the difference between background characteristics in the intervention and control group followed up at 24 months (after further dropout).
